# Psychiatrization of adoption practices in contemporary Poland

**DOI:** 10.3389/fsoc.2022.869593

**Published:** 2022-09-16

**Authors:** Anna Witeska-Młynarczyk

**Affiliations:** Institute of Ethnology and Cultural Anthropology, University of Warsaw, Warsaw, Poland

**Keywords:** adoption, Poland, psychiatrization, social policy, biogovernmentality, diagnostic cultures, psy-disciplines

## Abstract

In this article, I propose to take a closer look at the practices of kinning in the context of adoption in contemporary Poland. I am interested in the social production of this ‘unfamiliar kind of kinship' and the positions of various actors involved in defining the “adoptable” children and the “families of excess” capable of adopting. My focus will be on the ways in which the psy-knowledge and practices are implied in these social processes of defining and delimiting the norm, the proper, and the ideal. This process can be called a progressing psychiatrization of kinning, this time developing on a specific terrain of adoption (i.e., the most desired state of exception from ideal family—nuclear, heteronormative, based around married, and non-divorced couple). I will consider both top-down and bottom-up processes within which the individuals, state institutions, and psy-knowledge interact. Thus, I propose to look at a sub-process of psychiatrization, which takes place in the specific ethnographic context at the intersection of family and social policies, medicalization and psychologization of familial relations, and troubled, disconnected biographies. Throughout the article, I discuss how the adoptive families become patient-consumers within the system of healthcare. It is despite the fact that when they enter the adoption network, they start to take part in the political process of solving the social problem. In fact, they become a part of the network, which enables privatization of the social problem and works toward individualizing the responsibility for solving it.

## Psychiatrization of kinning

*The authority affects how we experience our bodies […]. It also affects how a society supports or fails to support our bodily suffering and struggles (Wendell*, *1996**, p. 9)*.

Beeker et al. (2021) invite social researchers to discuss psychiatrization of society. The term means the complex processes through which people's lives are being increasingly affected by psy-knowledge and practices (Rose, 1989, 1996). Psychiatrization, as diverse as it is, influences many aspects of contemporary human life. In this article, I will attend ethnographically to the ways in which psychiatrization alters the adoption processes in contemporary Poland.^1^

On a very general level, psychiatrization means a growing tendency to describe people's behaviors in terms of mental health, using cultural tools created not only by psychiatrists but also by psychologists, therapists, educators, or neuroscientists (Beeker et al., 2021). The notion of psychiatrization marks how the increasing amount of people's experiences is being observed, interpreted, and acted upon through language, concepts, and technologies, as well as by the institutional practices submerged with biomedical psychiatry (Coppock, 2020, p. 3). The expansion of psy-disciplines (Rose, 1989; Foucault, 2003) implies their entanglement in the new forms of government that no longer imply exclusively “disciplining” and “imposing from above.” Theoreticians speak about the new mechanisms of power (“biopower” or “biogovernmentality”) operating through dispersed networks or complex interconnections between sovereign, disciplinary, and biopolitical forms (Foucault, 2008), resulting in the internalized and embodied modes of managing the self (Rose, 1989). The responsibility is increasingly being placed in individual hands, making people manage their and their closed ones' wellbeing as if it depended on their own will and dispositions. People get access to medical categories by which they describe the states they are in. They further interpret what is going on in their families. They act upon the scripts they learn. Made widely available and legitimized by the state institutions and various psy-experts, the conceptualizations of mental health and specific problems defined within psy-disciplines have become the dominant, authoritative knowledge, which affects not only the way the body is experienced but also how the society deals with human suffering embedded in unequal social relations (Wendell, 1996). Psy-knowledge and practice mediate not only the management of ill-health but also the complex processes of kinning managed by the state bureaucracy. The lay appropriation of medical and psychological frames of reference and the material artifacts produced along these lines help the processes of psychiatrization settle down in the culture of everyday life, making the political aspects of social suffering less obvious. This is particularly the case when the psychiatric language and practice focusing attention on individual deficits infiltrate the public institutions responsible for managing lives of children taken away from their first families due to neglect, violence, or other serious breaches in practices of care. In such cases, the “psy” categories are being used to organize the politicized life of children.

Taking the unfamiliar kind of kinship (Carsten, 2004) as a topic, I aim to highlight the progressing psychiatrization of kinning ethnographically as it develops in contemporary Poland. I will consider both the material (e.g., the adoption centers along with the diagnostic apparatuses) and the ideational aspects of psychiatrization (e.g., the emergent definitions and therapeutic imageries), using top-down and bottom-up processes within which individuals, state institutions, and psychiatric knowledge interact (Beeker et al., 2021). My focus will be on a sub-process of psychiatrization that takes place at the intersections of family and social policies, medicalization and psychologization of familial relations, and troubled, disconnected biographies of children.^2^ Hence, in this article, I look at the order in the making as the kind of knowledge structuring the interactions concerned with foster care and adoption has transformed, and the new categories of subjects defined in the psy-language have emerged.

## The research

The work presented in this article is a part of a larger anthropological research project titled “Adoption as a process, experience and institution – an anthropological perspective” conducted by the Childhood Interdisciplinary Research Team at the University of Warsaw in Poland.^3^ The project commenced in 2018, and it was extended beyond the 3-year time frame due to pandemic. During the research, a group of anthropologists used a mixed methodology—participant observation, ethnographic interviews, discourse analysis, and the research techniques taken from the childhood studies—in order to explore the experiences of adoption in contemporary Poland. The aim of the project was to analyze adoption from the anthropological perspective, taking into account its complexity and diversity, as well as the voices of various actors involved in the process (candidates for parents, adoptive parents, adoptees, foster carers, and experts).

This article is based on some of the materials gathered during the project. In particular, I derived data from the ethnographic in-depth, open-ended interviews conducted in Poland with over 50 actual or prospective adoptive parents (mainly mothers), eight people involved in foster care, 18 young people and adults who were adopted and their siblings, and nearly 30 people working in adoption centers, in courts, in counseling centers, and as social workers.^4^ I further relied on the ethnographic fieldwork I conducted in institutions responsible for implementing the social policy in regard to children taken away from their biological parents. Moreover, I relied on informal conversations, observations, and fragmentary insights obtained in everyday situations. A significant inspiration for my reflections was from the autoethnographic group composed of the foster carers and adoptive parents that I had organized and run within the framework of this project (Witeska-Młynarczyk, 2022b).

The personal details of the people whom I, or my colleagues, talked to are fully anonymized. Some of the details contained in individual stories were changed so as not to allow for identification. Also, the details allowing for recognizing specific adoption centers and their employees were modified. For this reason, no specific geographical locations are mentioned in this article.

## The children-in-waiting

Currently in Poland, there are 64 operating adoption centers that connect the couples^5^ who want to become adoptive parents with children who were separated from their first families by the court decisions due to neglect, violence, or other serious issues. Each year, qualified couples adopt around 3,000 children (Wykonywanie zadań przez ośrodki adopcyjne, 2017). The average adoption process lasts 2 years—from the moment the candidates are registered as qualified till the moment the court issues a decision confirming adoption (Wykonywanie zadań przez ośrodki adopcyjne, 2017). The prospective parents may wait for the qualification and the training another 2 years. There are many more number of people who want to adopt than the number of children whose legal situation allows for adoption. Approximately 98% of children qualified for adoption get adopted (Wykonywanie zadań przez ośrodki adopcyjne, 2017). The majority of the employees in the adoption centers in Poland are trained as psychologists or pedagogues, so they assess the families and the children using their psy-competences. The main tasks of the institution are to qualify children for adoption, to select and prepare the prospective adoptive parents for adoption, to support women who want to give away a child for adoption, to manage the paper work necessary for the legal procedure of adoption, and to store the personal data connected to the adoption process. The prospective parents, when selected, are assessed by the adoption centers in terms of mental health, economic resources, and social networking.

The children are assessed as suitable or not suitable for adoption, as I understand from the interviews, primarily on the basis of their health, the ability to attach, and the possible existence of attachment to the current caregivers.^6^ The processes of psychiatrization and diagnosis are far from definite. They should be approached as emergent in the area of adoption in Poland. For example, the post-adoption support understood as a special category of social and medical service is almost non-existent in Poland. This is another field of psy-expertise, which may potentially grow. Some of the adoption centers offer therapeutic groups for adoptive parents or consultations, yet, these services are scarce, and many adoptive families are ambivalent about contacting the adoption centers in case of problems partially because they associate the institution with assessment and control. As argued by Frank Furedi, “the therapeutic culture conveys a strong sense of unease toward the private sphere” (Furedi, 2004, p. 66). The prospective adoptive parents we talked to in this research repeatedly reported that the interaction with “their” adoption center was strained. The main source of unease was suspicion they felt toward themselves and the fear dictating that it was better not to share everything. Eventually, when the court issues a decision constituting the new family, the adoptive family starts to function “as any other family.” Hence, any support sought for by the families in the post-adoption phase is supposed to be a part of the non-specialized, both public and private, system of mental healthcare.

Foster care encompasses various care arrangements, which differ from adoption. In case of adoption,

“The privacy of the adoption family is prioritized and protected while no contact is maintained with the first family. The new birth certificate replaces the first one and the previous last name is overwritten by the surname of the adoptive parents. The adoptive parents can also change the first name of the child if they wish to do so” (Maciejewska-Mroczek and Witeska-Młynarczyk, 2021, p. 78).

The foster care is organized in forms of family-like (related and unrelated, professional and non-professional) and institutional care running under the auspices of local authorities. These forms of care differ in terms of benefits, access to special educational programs or supervision, or the fact of biological relatedness with children. Unlike in the case of adoption, within these arrangements, children normally maintain contact with their first families. They keep their names, and they gain no rights to succeed from the foster carers.

Currently, in regard to the first families, the Polish state promotes the reintegration policy. When families are spotted by social workers as in need of intervention, among others, due to bad care provided to children, the state invests in supporting them so that the children could be reintegrated with their first carers and properly cared for in their initial homes (Dzieci sie licza, 2017). Despite such defined strategic and ideological aim, the number of court decisions to separate children from their families for the reasons of bad care, neglect, or insecurity grew from 4,400 decisions in the year 2000 to 10,675 decisions in the year 2015 (Dzieci sie licza, 2017). The majority of children whose parents were recognized as unable to perform their parental obligations and rights live in some form of foster care, including the care provided by the near of kin. In 2015, the number of young people in this situation reached 62,036. In 2015, 2,947 decisions about adoption were issued by the courts, including 199 adoption arrangements according to which Polish children were sent abroad (Przysposobienie w latach 2000-2018 oraz w pierwszym półroczu 2019, 2019). The remaining children were adopted within the country by the families which met the criteria and were selected by the adoption centers. Hence, the children qualified for adoption constitute a small fraction of the larger group of children somehow diagnosed by the system as being endangered by their familial environments. In the years 2015–2017, the children qualified for adoption transited to adoptive families from the family foster care (3,469), biological families (1,326), and institutional foster care (1,214; Wykonywanie zadań przez ośrodki adopcyjne, 2017, p. 11).

I propose to approach this group—the children separated from their first families, as yet another category of children-in-waiting (Witeska-Młynarczyk, 2020).^7^ I paraphrase here Timmermans and Buchbinder's term “patients-in-waiting” used “for those under medical surveillance between health and disease” (Timmermans and Buchbinder, 2010, p. 1). According to these authors, the patients-in-waiting inhabit a liminal state between pathology and normalcy. As argued by Maria Liegghio, psychiatric knowledge and practice rely on this binary opposition of normal/abnormal, and the diagnostic process is meant to mark the individual as healthy or unhealthy (Liegghio, 2016, p. 114). In this article, I will describe the many shades of the processes of defining and diagnosing implied in the adoption practices. The psy-language has been increasingly used to define the life situations of the Polish children and the regime of state-involved care they encounter. I will point to the ways in which the process of diagnosis is being scattered and delegated to the non-medical institutions managed by the state, composed of people, to various extent, educated in psycho-disciplines. Lingering on the moment of transition (a child/prospective parents are to be qualified for adoption), I highlight the social and political practices of delimiting the normativity implied within the adoption practices formulated as “family of excess,” “adult who coped with loss well,” or “adoptable child.” Patients-in-waiting inhabit a liminal state between pathology and normalcy. The children taken away from their first families are marked by “the extraordinary conditions” (Jenkins, 2015) and experiences, and they are waiting for a possibility of entering the “state of normalcy,” which is imagined as a movement of joining the chosen “families of excess” (the emic term used by the people working in the adoption centers for marking the selected prospective adoptive parents who have more than enough). The prospective parents, most commonly dealing with the issue of childlessness and hence touched by a psychological notion of loss (the state of which is also being assessed by the adoption center), are meant to create a proper family, which, in the case of adoption, is increasingly conceptualized as reliant on the psychiatric and psychological help in order to heal the trauma understood as an integral part of the child's biography.

## The biopolitical bureaucracy

The analytical focus on adoption practices allows for capturing the processes of expansion of the psy-complex beyond the medical space. In particular, it is interesting to observe how the new psy-conceptualizations are interwoven with the politics of the state on a microlevel. Nissen and Bech Risør (2018) used the concept of “biopolitical bureaucracy” in order to highlight the multiplicity of human and non-human actors involved in the processes of medical diagnoses. I see adoption centers and other institutions involved in the practices of adoption and foster care as constituting biopolitical bureaucracy, yet I locate them at the margins of the medical practice. At the same time, I recognize them as central for defining the adoption stage in psychiatric terms. The entire network of institutions and knowledge that work toward the assessment and elaboration of children's and candidates for parents' subjectivities and their destinies are increasingly reliant on the psy-knowledge and practice. Howell (2006), who researched the adoption practices in the United States, proposed a similar term—the “psychotechnocrats”—to highlight the influence of psy-language and practices undertaken by the state officials working in such institutions as adoption centers for the intimacy of children and their carers. The state employees, relying on the various regimes of knowledge (psychological, psychiatric, technocratic, neurological, economic, etc.) and on the socially accepted values, assist the carers in producing the imaginarium of good care. This may include the differentiation between the temporary foster carer and an adoptive parent as different types of carers who are meant to generate different types of attachment with children, or more specific elaboration on ways in which to handle children's past. Along the same line, Brunila and Lundahl (2020) argued that the politics of therapy represent a new form of biogovernmentality as they link the individual subjectivities with the state policies. In the case of adoption, the politics of therapy imply finding families that could perform a therapeutic work for children with adverse childhood experiences while acting as a regular family. Through the trainings and other practices, the psychobureaucrats assist in the carers' efforts to become successful caregivers (Krawczak, 2022). The carers are set in an interactional framework with the publicly formulated expectations each time they try to perform good care for their children (Roux and Vozari, 2017). The adoption centers have been gradually integrating new psy-knowledge into practice. From the interviews, it seems that the psychiatrization of adoption practices should be considered not only using a top-down but also the bottom-up process in which non-governmental actors played an important role of popularizing the new knowledge about adoption and demanding change.

## Capturing the change

“[…] ‘science' doesn't have the power to impose itself. If it spreads, this is because there are actors outside the laboratory who associate themselves with it. And they may pick through what is on offer and take bits and pieces. They do not get overwhelmed by a massive structure or a coherent episteme” (Mol, 2002, p. 64).

Poland supports the “closed” model of adoption (see Maciejewska-Mroczek and Witeska-Młynarczyk, 2021), which means that the privacy of the adoptive family is prioritized over the right of children to know their roots or the first family's right to maintain contact with the children. Until recently, adoption has been typically kept secret within the families, in particular the children often were not informed about their past and about the fact that they were adopted until they reached adulthood, which added to the culture of secrecy (Maciejewska-Mroczek and Witeska-Młynarczyk, 2021). The employees of the adoption centers talk about the late 1990's as the period when new knowledge and practice started to permeate their professional circles. Among others, the centers have gradually introduced the elements of advice on how to talk to children about their past to the training program.^8^ Also, the knowledge about specific diagnoses like FAS, ADHD, and RAD started to circulate. The attachment theory, including the knowledge about the attachment styles, took the central stage. In fact, what happens with the knowledge conveyed by the adoption centers is completely up to the adoptive family according to the family's right to privacy. The adoptive families have a large pool of sources of psy-knowledge not connected with the adoption centers like non-governmental organization, other professionals or the social media. I suggest that one of the ways in which the largely unknown past is managed and tamed by the newly constituted families is through therapeutic interventions and the focus on the bodily manifestations of the past in the present. These are being named in the psy-language in a form of diagnosis like attachment disorder. This phenomenon could be named a psychologization of the embodied past.

The character of adoption in Poland has changed over the years. The cultural transformations influencing adoption practices are manifold, and the encroaching psychiatrization is entangled in the more complex societal processes including normalization of single motherhood; opening of the public debate concerned with the reproductive rights; the easier access to new reproductive technologies; encroaching culture of confession and therapy; reconfiguration of family dynamics; the increased significance being given to children, their rights, and their psychological wellbeing; the growing professionalization of state bureaucracy; the increase in transnational flow of psycho-expertise, knowledge, and practice; and the lessening of the tabu posed on family violence. Marlena, a women in her 40's, a manager of one of the adoption centers in Poland explained to me the practical difference that had unfolded during her career:

Marlena: At the beginning of our work these were mostly newborns left by single mothers in the hospitals. I talk about the majority of adopted children. Meanwhile now, we hardly meet these kind of mothers. The children we deal with now have been in the foster care for some time already and their parents or caregivers were deprived of their rights to care. Most commonly, these children have experienced all kinds of violence or serious neglect in the critical 1st years of their lives.

This observation points to the crucial qualitative change that took place within the field of adoption. Adopted children are now being recognized as marked by the adversary experiences definable in the psy-language and treatable through therapeutic conceptualizations and techniques. This means that a larger and more complicated network of actors, definitions, and discourses have become involved in the process of separation of children from their first parents and the acts of relating them to unfamiliar adults.

The children meant for adoption are more and more frequently talked about by the employees of the adoption centers as “traumatized” early in the prenatal period and in the initial years of their lives:

Zuzanna (a manager of the adoption center): But the majority of these children come from alcoholic pregnancies, so, basically, during the entire pregnancy they lived through trauma and stress. They were exposed to violence, because the alcohol consumption during the pregnancy should be considered an act of violence against children. Additionally, they were exposed to the results of bad treatment, also when the children were taken away from their family houses by the means of police intervention.

At the same time, as expressed by Mirka, another manager in one of the adoption centers in Poland, and this information came up repeatedly in the interviews with other employees of adoption centers, the expectations of the candidates for adoptive parents are quite unified:

Mirka: The most common expectation is that the child will be healthy and young.

In this situation, there is some work of elaboration going on in the realm of expectations and understanding the supply side in the adoption process. Since the adoption centers have less and less newborns and they are increasingly aware of the extent of the “invisible disabilities” (Blum, 2015) the children qualified for adoption embody, and the scant diagnostic and therapeutic possibilities they have, they take on themselves the task of “enablement” (in polish *urealnienie*) *vis-à-vis* the prospective parents. This emic term used by the psychobureaucrats conveys the desire on the side of the state employees to make the prospective parents aware of the type of children currently available for adoption. Kasia, a psychologist working in one of the adoption centers, said about the prospective parents:

Kasia: Well, they are more realistic now. They used to think - *this is a poor child of well- educated parents, who died in a car accident, a blond girl with blue eyes*. […] Now, the parents know how the child gets into the system and what are the possible reasons for the biological parents not to be able to take care of the child. So this is changing.

## Individual awareness and the diminishing ethos of public responsibility

The prospective parents' awareness of the possible/uncertain disorders to be treated in future is being developed during the trainings provided by the adoption centers. At the same time, there is a lack of solid diagnostic work prior to adoption, and there is no decent post-adoptive support provided by the state. As argued by Frank Furedi, one of the defining features of the therapeutic ethos is “awareness” (Furedi, 2004, p. 73). To be aware of the correlations between individual and family pathology means gaining an insight into the ways in which mental health issues are managed (Furedi, 2004, p. 76). The awareness of the connection between the child's health and the context of its first family is built by the adoption centers throughout the training sessions prepared for prospective parents. Eventually, the new parents are imagined as a “therapeutic” family for the adopted children and the main guarantee of the wellbeing of adoptees. They are chosen as capable of helping the children in lifting up the trauma.

From the economic point of view, adoption is the cheapest option for the state that is responsible for the wellbeing of its children-citizens.^9^ Talked about by the employees of the adoption center as “a miracle,” the best possible option the child-in-waiting can dream of, marks the decline of an “ethos of public responsibility” (Furedi, 2004, p. 72). The adoptive family is imagined as the one that has the resources (both economically and emotionally and as educated and aware citizens) to take the individual responsibility for the child's transition into “normalcy.^10^” The intensive education of candidates for adoptive parents on the theme of possible disorders increases their awareness and promotes their urgency for self-diagnosing and organizing therapies. When the adoption process is complete, the adoptive families become consumers “who actively seek out diagnosis and treatments based upon their self-assessments of symptoms” (Ebeling, 2011, p. 826). They take on themselves the sole responsibility for the stumbles (Witeska-Młynarczyk, 2022b). The adoption centers perform the work of preliminary diagnostic practices mainly by increasing the awareness of disorders. As such, I recognize the adoption centers as the “brokers for psychiatrization.^11^” Psychotechnocrats (Howell, 2006) play a role in the preliminary diagnostic work (Dew and Jutel, 2014) as “disease-spotters” (Philips, 2006, p. 434)^12^—that is, the initiators who push families onto their diagnostic journeys.

Mirka: We say: “unfortunately, our children are like this, you have to accept the possibility that something will go wrong.” They [the parents, AWM] start to open themselves for this. However, a child with some evident disabilities have no chance for being adopted […]. Yet, even if there is no FAS diagnosis, there is a really big chance that something will be wrong because the mother was drinking alcohol during pregnancy.

Changes in the imageries around adoptive kinning have been gradual. The pivotal moment that psy-technocrats point to is the year 2000, when the new knowledge started to infiltrate the circles of psychologists and social workers. At this point, prospective parents are confronted during the trainings with very specific diagnostic knowledge. The disorders discussed can be enumerated. The pedagogue working in one of the adoption centers in Poland narrates it in the following way:

After the year 2000, there begins the knowledge about FAS (Fetal Alcohol Syndrom), FASD (Fetal Alcohol Spectrum Disorder), RAD (reactive attachment disorder), traumas. The trainings for the foster care called PRIDE began. The standard of knowledge was imposed by the people from the Association Our Home. There is a number of projects, for example Martynka's Friends' Foundation—they take a lot from Italy, from the USA.

The flow of knowledge influencing the adoption practices has been mediated by various bodies, including non-governmental organizations or professional associations. As such, the psychiatrization of kinning is advancing by both the bottom-up and top-down flows of knowledge, which is transnational in its nature.

Biomedicalization, or, to be more precise, psychiatrization has become increasingly relevant in the case of adoptive families as the health of the adopted child marks the adoptive family's success or failure. Child's health and the quality of attachment itself become a commodity (Clarke et al., 2003), a condition (e.g., a proper attachment) which is sought for, something that has to be maintained or rather actively produced after the child is adopted, among others, by the reliance on the psy-knowledge and help of the experts.

The adoptive families are increasingly being imagined as in need of assistance in the process of working out proper family relationships. On the one hand, they are meant to be like any other family, yet, at the same time, they are imagined as a special kind of family (Maciejewska-Mroczek and Witeska-Młynarczyk, 2022).

Agata, an adoptive mother of a girl, in her 40's, who has actively searched for possible pieces of training and workshops during which she and her husband could have worked on their attachment styles and readiness to emotionally support their child (despite the fact that the girl holds no diagnosis), actually sees the training provided by the adoption center as useful:

Agata: It [the training, AWM] helps to recognize certain behaviors. If something is happening, it has a cause, so, we shall be aware of it. Trauma, because different behaviors come as a consequence of trauma and different as a result of the attachment disorder, so, this is… […]. It helped me a lot. And there were some embittered voices hearable [of the other adoptive parents—AWM] complaining that this was insufficient [the knowledge conveyed during the training in the adoption center—AWM]. That the issues were merely signaled. But I agree that there actually was not enough space during the training. And apart from that, as far as you don't find yourself in some specific situation, it remains a theory.

As noticed by Roux and Vozari who conducted research with adoptive parents in France in the context of contemporary adoption, it is not enough to be a parent; one has to become a very particular caregiver. In such sense, the discourse of adoptive parenting should be considered as something more than a moral discourse. It becomes an instrument of power (Roux and Vozari, 2017, p. 13). According to these authors, the institutions of social care promote the particular kind of ethics, the one which implies autoregulation, the constant effort of improving oneself. Adoption processes serve as a lens through which we may observe the contemporary forms of political power and the ways in which the socially situated actors interact with them (Roux and Vozari, 2017, p. 19). The state remains a pivotal regulatory actor of the family life, even when immersed in the network of non-governmental and private bodies. “Therapeutic governance” represents a new form of governmentality (Brunila and Lundahl, 2020) as it links the practices of constituting the individual subjectivities along with the ways in which the state functions. It is relevant in this context to take a look at what is stated as good, true, and desired in the practices performed by the state institutions (Brunila and Lundahl, 2020).

In the course of the adoption process, in particular during the training offered to the prospective parents by the adoption centers, the “diagnostic power is removed from the exclusive purview of medical authority” (Ebeling, 2011, p. 831) and placed in the hands of psychotechnocrats; such arrangement opened up a space for negotiation and meaning making invoving many actors and streched in time; the expectations put on adoptive families to become therapeutic families generate the feelings of anxiety and an immense effort put in trying to succeed to rescue the child. The parents are made to believe that the result depends solely on their efforts (Witeska-Młynarczyk, 2022b).

## Seeking for normalcy

While the adoptive parents are made aware of the uncertainty of children's health, they are educated in the possible psychological interpretations of the problems they may encounter after adoption, and there actually is no demand for disabled children or the children with serious medical diagnoses, including psychiatric diagnoses. The situation of adoption is still highly marked by the expectations and desires to become “a normal family” (Maciejewska-Mroczek and Witeska-Młynarczyk, 2022). Mirka, a manager of the adoption center in Poland, notices:

Mirka: Certainly, there is no openness for disabled children and such children are more and more numerous.

[…]

Researcher: Does it often happen that the candidates indicate the readiness to adopt a disabled child from the very beginning?

Mirka: These are extremely rare situations.

The “invisible disabilities” (Blum, 2015, p. 42–50) become a non-human actor shaping the processes of adoption relying on some forms of diagnostic work. According to Linda Blum, the term “invisible disabilities” means neurodevelopmental disorders that are not immediately noticeable and more difficult to diagnose than the physical disability. This lack of visibility opens up the field of anxiety, the unknown, but it opens up the space for hope and political game. It is particularly so in the case of attachment as it is understood as a relational thing, depended upon two parties. In the practices of adoption, we have interwoven the contemporary version of the myth of control. As argued by Susan Wendell, what comes along with it are the burdens of blame and guilt that are fostered by the myth (Wendell, 1996, p. 9). At the same time, the responsibility is transferred from the state to a single family. This transfer of responsibility was narrated by Marlena, a manager of adoption center, during an interview:

Researcher: Well, and another challenge for the adoptive parents is the reactive attachment disorder. Is that correct? Do you diagnose it in children?

Marlena: We have to state our opinion about it and, during the training, we are preparing the candidates for it.

Researcher: Aha.

Marlena: For these reactive attachment disorders, it seems to me, they are prepared to deal with those. I also think that, even though there are no research results to rely on, with such a wise, therapeutic approach of the adoptive parents, well, this is the kind of thing that they are able to fix.

Unlike Marlena, many employees of the adoption centers we talked to recognize the child suffering from the reactive attachment disorder as unsuitable for adoption. I suggest that this collective reflection results from a recognition of the demand side and the actual unreadiness of the majority of the prospective parents to build a family which is not “like any other family” or which becomes a family for an older child or siblings. When I asked a psychologist and a pedagogue in one of the centers whether there are children who are “unadoptable”, at first they said:

Almost each child younger than 18 is adoptable, but a 17 years old and disabled female teenager actually is not.

In a further conversation, they explained that those children who are adoptable actually show an ability to attach to another person. They expressed uncertainty about the ability of children suffering from FAS to develop attachment. In fact, they voiced their concern about the actual possibility of diagnosing children. It is so because the problems are imagined to be located in the brain—“but, physiologically, on the level of the brain, whether the child will be able to develop attachment, we do not know it. It will develop a different kind of connection,” they stated (from fieldnotes). I understand it as a commentary on the condition of the diagnostic uncertainty and the actual inability of the state bureaucrats to tame and understand and reliably communicate the children's biographies inscribed in their bodies. Their structural position is ethically difficult, and they try to navigate uncertainty by reference to the psy-knowledge, which gives some possible answers and refers to medical authority, yet considering future development of a child.

## Toward definitions

In the specialized psychiatric literature, both adopted children and children in foster care start to be recognized as separate subjects worth attention due to problems with mental health. Such formulations are relatively new in the Polish medical literature (Szmajda and Gmitrowicz, 2018).

Szmajda and Gmitrowicz (2018) argue that children and adolescents reared in foster care more often than their peers brought up in two-parent families suffer from self-injuries and make suicide attempts. They notice that the average age of psychiatric diagnosis in such children is lower than that in the rest of the population, and they are more often hospitalized. Hence, institutional care, including family foster care, is recognized as a risk factor for children (Szmajda and Gmitrowicz, 2018). Pawliczuk and Kazmierczak-Mytkowska (2014) is cited as the only research conducted in Poland on this topic with the conclusion that over 50% of children reared in institutional care suffer from mental health problems. This includes psychiatric diagnoses. This argument strengthens the imagery of adoption as an ideal place. Yet, some adoptive families we talked to turn attention to difficulties they encounter every day. Marta, an adoptive mother of two girls, was apparently not ready to take the entire responsibility for the struggles that came along with adoption:

Well, so this training [about the training provided for the candidates for adoptive parents]… no one prepared us for the kind of problems we encounter […]. We reflected together with my husband that if they had told the people, how much would the adoption rates fall? I think it is better for adoption rates to fall and for the people to be prepared and for the adoption to bring about good results. And instead, we are tired, our frustrations are being transferred on the kids. Because sooner or later this is what happens. And you have no chance to avoid it.

The image of adoption as a struggle is rarely evoked. The Internet opened up the space for discussion on the fora run by non-governmental bodies like Nasz Bocian—an association meant to provide the professional support for the people coming to terms with childlessness; yet, while lifting up the sense of failure it still strengthens the individual efforts focused on providing the proper care. The myth of adoption as an ideal solution, a miracle, and as something that can be controlled through therapies predominates, and the alternative narratives rarely see the public light (see Janus, 2022; Potocka, 2022). Failed adoptions are spoken about rarely. While the official number is lower than one percent of adoptions each year, that is, adoptions which are legally dissolved, many adoptive relationships remain seriously strained (Janus, 2022; Witeska-Młynarczyk, 2022a).

The psy-experts start to recognize the struggle by defining the adopted children as another group worth a systematic study and focus. Skiepko and Bra̧goszewska underline that “adopted children, in comparison to the children brought up in biological families, constitute a higher proportion of patients appearing at the psychiatric consultations and being hospitalized” (Skiepko and Bra̧goszewska, 2009, p. 207). The crystallization of the category of adopted child in psychiatric discourse and practice may further influence the adoption practices. The adoptive parents are meant to fulfill the role of a therapeutic parent, which becomes a measure of success for the adoption project. Successful projects will need a professional ally. A troubled child will be the focus here.

## Practicalities of care

Social practices of defining children suitable for certain types of care take place within the institutional walls, where state bureaucrats have a chance to collectively reify the reality which they face every day. As stated by Susan Wendell, “Questions of definition arise in countless practical situations, influence social policies, and determine outcomes that profoundly affect the lives of people with disabilities” (Wendell, 1996, p. 11). The following is the fragment of my fieldnotes illustrating ways in which the social workers negotiate order by relying on the psychiatric vocabulary.

### From fieldnotes

It is April 2019. I participate in an assembly of the local family centers (Powiatowe Centra Pomocy Rodzinie, PCPR). The manager of the regional office for social policy (Regionalny Ośrodek Polityki Społecznej, ROPS) agreed for me to take part in the meeting. ROPS manages the institutions responsible for the implementation of social policy created for the families and children. It further supervises the adoption centers. PCPR is an institution that supervises the foster families. Before the year 2011 (the law was amended), the foster families were cooperating with the adoption centers. Now, the adoption centers specialize in adoption only. During the meeting, one of the representatives of the local family center notified she wanted to have a voice. She started to refer a problem which she classified as “children with opinions” or “children with psychiatric diagnosis.” She referred that in the voivodeship there were 399 children with such diagnosis in foster families, including 114 in the institutional care. She described this as a “new terrain,” “a recent problem” dating a dozen or so years back and that the largest group of children with diagnosis can be found in the institutionalized care. In her short statement, she made a reification by distinguishing between “the children with psychiatric problems”—defining them as those whose behavior is disorderly and “the children with pedagogic problems” or various problems appearing in the practices of care. She suggested for the children with psychiatric problems not to be placed in the foster care. As an example of a child with psychiatric problems who should not be placed in foster care she brought about a story of a 14 years old girl, who had attempted suicide and who had sexual contact with adult man and the court decided to place her in the foster care. “This kind of child should not be placed in the foster care”—exclaimed woman in a concerned tone.

In this social situation, there emerged a category of children who do not fit foster care. The local bureaucrat, in her speech act, proposed to use psychiatric diagnosis as a way to identify children who should not be embraced by a certain kind of care. It was said, but, I assume, she meant such children to be meant for hospitalization. Here, we have a social attempt at identifying a micro-process of negotiating order with a usage of the psychiatric apparatus. My feeling is that the women made practical distinctions between more and less valuable lives using the psy-language (Judith Butler in Gessen, 2020). This ethnographic example pictures a micro-movement that may gain no larger relevance; however, it tells about the presence of the psy-language. It is illustrative of the ways in which the psychiatric knowledge, taken away from the medical context, is being used by the state officials to order reality, to categorize children with an aim of organizing care for them. It is typical for psychiatrization movement outside the medical space. It implies the merging of the language of psychiatry with governmentality practiced by the state apparatus in the field of social policy. Eventually, it may be considered as part of the process of “vulgarization of psychiatry.”

## Diagnosing the ability to connect

One of the elements of the encroaching process of the psychiatrization of kinning is that children are stratified based on their abilities to connect. The imaginary of their abilities is now being fed by new neuroscientific and neuropsychological discourses and research, as well as it is based on the constantly developing attachment theory. On the brain level, they may be unable to form the kind of attachment that is imagined as proper for the adoptive family. They may form other kind of connections (not attachment) that does not fit a model of an adoptive family, which is to imitate a “normal” family. At the level of the state institutions like adoption centers, complex processes of elaboration are taking place. These processes imply categorizing children suitable for adoption based on their medical condition and the demand side.

When the parents of the children placed in the temporary foster care are being deprived of their parental rights by the court, the children are marked as “legally free” and they may be considered for adoption. If such children appear in the system, the employees of the adoption center need to gather information about them. Marta, a psychologist in one of the adoption centers in Poland, describes this process as scattered among many actors and material objects. From her position, the difficulty is to rely on the information given by another institution and passed on paper:

Marta: Gathering information, completing the history, about the first family of the child, it requires a lot of effort from us, and, actually, much trust being put in the people who generate this knowledge, that they will provide us with satisfying set of documents. The prospective parents will ask questions.

Researcher: Can you explain in more details about how the information is gathered?

Marta: It is all described in the legal act.

Researcher: Ok, but apart from being described legally, there are people who gather the information and pass them on. How is the information passed on?

Marta: On paper.

Researcher: So you mostly deal with the information passed on paper.

Framing the children as adoptable is an action taken jointly by many different actors: social workers, judges, foster carers, employees of the adoption center, the diagnostic articles prepared by psychologists or psychiatrists, and many others, like buildings, technologies, and knowledge. The categorization does not come as a discrete act. It rather emerges through the actions taken by various institutionally affiliated people located in various spaces and acting upon certain ideas of children's interest, proper care, or proper diagnosis (Witeska-Młynarczyk, 2018).

In order to get to know the children's situation in more detail, the employee of the adoption center needs to require information from the institution managing the foster care. The documents may include a psychological diagnosis, an opinion about the child, and the social worker's opinion about their first family.

Researcher: So, the child is diagnosed.

Marta: Well, the institution supervising the foster care sends us the information. […] Depending on whether they already have it or not, they make an assessment of the child's situation and they send us the complete files. Or, like the last time, I replaced my colleague, they sent one document and all the others were missing. […] So, everything depends on the institution which manages the foster care.

Marta expresses her feeling of lack of trust toward the competences and reliability of other institutions she cooperates with in the adoption process. Once the files are complete, the employees may go to see the children and proceed with their own diagnosis. Marta's colleague—Kasia—explains:

Kasia: It may happen that we make our own diagnosis. If the diagnosis sent by the-

Marta: -by the organizer is-

Kasia: -is insufficient.

Marta: Of low quality, so-

Kasia: It does not meet our expectations. […] So then, apart from the conversation, actually, there is an element of the diagnosis in this meeting, meaning observation, sometimes it even implies a diagnostic test, like developmental.

Researcher: What happens when you see that the child perhaps needs a deeper neurodevelopmental diagnosis? Does it happen? What can happen in such situation? Can you demand such a diagnosis from the carer?

Kasia: On the stage of qualification well, my opinion is that in Poland children, or at least here, children are underdiagnosed or very badly diagnosed. A neurologist writes that everything is ok, while it is not ok. So, the level of diagnosis is low.

A diagnosis is often understood as a critical moment leading to a healing procedure. It can be understood as a term or a category that puts the world in order. You get to know that your children are suffering from adverse childhood experiences. Yet, the diagnosis can also be understood as a process (Jutel, 2018). It is increasingly talked about not as an act but as a “diagnostic work” and as a “disorderly process” (Jutel, 2011; Goodwin and Mc Connell, 2014; Nissen and Bech Risør, 2018) engaging various actors, things, ideas, and places. It implies “doing” (Mol, 2002) also performed in non-medical spaces and shaped by expert and non-expert voices and judgments (Büscher et al., 2010). Nissen and Bech Risør note that:

“Processes of a diagnosis include any activity surrounding investigations, assessments and negotiations pertaining to clinical and non-clinical judgments of ill-health. Different actors with their skills, experiences and sensing bodies are involved in these processes, in conjunction with technology and instruments of measurement. Studies of such processes have explored the enactment and the making of a diagnosis with particular focus on subtle intersubjective processes between health professionals and patients” (Nissen and Bech Risør, 2018, p. 15).

In the adoption network, adoptive parents are well-rooted in the social networks discussing the adoption process and the psychiatric knowledge related to it. Adoption centers are institutions devoted solely to the selection and training of adoptive parents, as well as to pairing children and parents. These are interwoven into other institutions of social care responsible for managing the first families and children taken away from them.

Both children taken away from their first families and the foster and adoptive carers are increasingly exposed to medical knowledge and practice both through their involvement in the biosiocialities, the expert discourse circulating in the popular media, and through the contact with the state officials who supervise and select them. People working in the adoption centers become agents of psychiatrization, yet their role in diagnostic processes varies. The adoption centers educate the prospective parents about FASD and RAD, yet most of the time, they face the lack of specialists prepared to diagnose small children. In addition, the actors involved in the interim care for children sometimes are inconsistent in taking responsibility for the diagnostic process. Another thing is the accessibility and financial availability of the diagnostic processes. There is also a conviction that children would not be adopted if a FASD or RAD diagnosis is given, which brings about ethical dilemmas into the every-day life of social workers. Because prospective parents are assessed by the adoption centers, they most of the time do not feel they can demand transparency or quality information (including medical information). In the interviews, they reported feeling impeded by the fact that these employees of the adoption centers eventually decide upon them gaining a possibility of adopting. They know they function as an element of the economy of lack, and they recognize the game is to be played carefully with those who are in the position to decide.

## The economy of lack

The contemporary adoption scene in Poland undergoes the process of transformation and should be recognized as the economy of lack, that is, the demand for a particular kind of children is much higher than the supply of, what I will call, adoptable children.

Marlena (the manager at the adoption center): At this point, I can say for now, for the 2019, that the most difficult is this knowledge that people have basically no chances for adopting. So this is an absolutely hopeless situation, and the fact that we are doing our job nonetheless—we train them, we support them, but the perspective for them to become parents is so far that, in my opinion, we could say it is unreal. And it will probably be the biggest problem… their anger.

Martha (a manager of the adoption center): Those candidates who are waiting the longest, they are from 2014th.

(Researcher): Oh.

Martha: They are the ones who came [to the center] in 2014. It is 5 years now, so it is a lot. Anna: Oh. And how many people do you have on the waiting list?

Martha: Like 25 couples.

The supply of “adoptable” children is not sufficient for those waiting. Despite the discourse of “the best interest of the child” (Maciejewska-Mroczek and Witeska-Młynarczyk, 2021), the system caters for the needs of candidates for adoptive parents. The employees of the adoption centers during interviews often explained how they would protect the parents by not offering them more than one child as they would not cope with more. During the interviews, we also heard about the siblings separated and given to different families, which is a straightforward expression of favoring the rights of the prospective parents over the best interest of children. Among other, the psy-knowledge plays an important role in defining adoptable and non-adoptable children, that is, it becomes a crucial ingredient of the dividing practices performed under the auspices of the state.

Hence, what has been happening with the influx of knowledge and the new diagnostic possibilities is a set of redefining practices, which work toward delimiting what is possible within the adoption process. What comes as a result of these tendencies is, I call, the tightening of the adoption system.

Adoption works toward reproducing the social hierarchies between the deserving adoptive family and the unsuitable providers of children (Briggs, 2012). As argued by Leinaweaver, to frame adoption by the rescuing metaphor (in Poland, the metaphor of miracle is more commonly used), blocks the possibility of a critical discussion about the social inequalities and the situation of the families from which the children are taken away (Leinaweaver, 2018, p. 9) and the children themselves. Adoption understood as an act of mercy—a miracle that takes place, thanks to the practice of unconditional love or deep therapeutic work—silences different shades of this process, that is, adoption experienced as a challenge, as an “epistemic struggle” (Jenkins, 2015)—both for a child and for the parents (see Potocka, 2022), as well as the first parents, whose rights are recognized only as long as the policy of reintegration is considered.

Brunila and Siivonen (2014) pointed out how neoliberalism (understood as a political ideology and a way of governing by the reference to individual rationality, freedom of choice, and attachment to the market) facilitates the spread of therapeutic cultures focused on improving psychological and emotional vulnerabilities ascribed to persons (Brunila and Siivonen, 2014). The therapeutic cultures and the focus on the individual feed well into the biomedicalization of human life. We recognize the increasing amount of problems encountered by a human being in the life course, as assessed as a medical issue possible to be solved through therapy (Nowakowski, 2015, p. 52–53). The lay people and experts in various cultural contexts align themselves with the processes of medicalization in order to meet their needs and cultural expectations.

## The political stake

As argued by Wendell,

Disability is socially constructed by such factors as social conditions that cause or fail to prevent damage to people's bodies; expectations of performance; the physical and social organization of societies on the basis of a young, non-disabled, “ideally-shaped,” healthy, adult male paradigm of citizens; the failure or unwillingness to create ability among citizens who do not fit the paradigm; and cultural representations, failures of representation, and expectations (Wendell, 1996, p. 45).

Constructed as they are at the moment, the adoption practices in contemporary Poland should be recognized as social conditions that fail to prevent damage to people's bodies. Among others, the expectations of normality, the ideal, play a role here. Marlena's words illustrate this imagery of the adoptive parents as “ideally shaped” citizens:

Researcher: How many of these families, do you think, would require some kind of psychological or therapeutic help later on?

Marlena: I think not many. […] A few years ago we came up with a motto that we are to prepare them in such a way so as not to face them coming back. We are to work with them in such away so as to make them aware, conscious and ready, so that they have such resources. And this is the idea imprinted in the law.

Following the contemporary practices of adoption in Poland ethnographically, I suggest that the Polish state, through the acts performed by the psychotechnocrats, works toward distancing itself from responsibility for the children-in-waiting described as “adoptable.” The adoption process is an integral part of the larger social project of stratifying children-in-waiting using psy-knowledge. The dividing practices reliant on the psy-language allow for distinguishing different kinds of children meant for different kinds of care. The distancing from responsibility is possible by reference to the family's right to privacy, the pursuit toward “normalcy,” and the realization of “the best interest of the child” envisaged as a placement in a nuclear, heteronormative family run by a well-selected and diagnosed married couple. By educating the prospective parents about the social milieu from which the children available for adoption currently come from and teaching about the findings in neuropsychology and trauma studies, the employees of the adoption centers hint “at something” while leaving ambivalent and unclear whether children are actually sick and, if so, what their sickness would entail (see Timmermans and Buchbinder, 2010, p. 417). The uncertainty of the state of child's health results from the insufficient diagnostic infrastructure,^13^ the actual lack of interest of the state representatives to perform a proper diagnosis in light of the fact that the prospective parents seek for healthy and small children (a proper diagnosis would entail the risk of lowering the demand side), and the tendency to lower state's costs (and in fact the failure to provide with the proper medical assistance for the children in foster care).

These structural conditions are imposed on prospective adoptive parents who desire to adopt a child and whose ideal model of life is a nuclear, heteronormative family in which a child develops from early years. In these conditions, a generic uncertainty is being produced (the unknown state of a young child who will be managed and taken care of by the new parents). I suggest to treat it as a by-product of the logic of state policy, which works along the economic rationality of demand and supply intermingled with the conservative ideology favoring the imagery of a nuclear, heteronormative family as an ideal place for a child to develop and distancing from responsibility. The state bureaucrats discipline the adoptive parents to take individual responsibility for diagnosing and going through numerous therapeutic interventions meant to turn an adopted child into an expected citizen with little costs on the side of the state.

## The private solving of the social problem

The new knowledge generated in neuropsychiatry and trauma studies help define a group of children whose adverse childhood experiences make them prone to being narrativized as in need of healing relationships, possible to be provided only by the idealized nuclear family who is well chosen—resilient, economically well-off, with a proper approach—“a family of excess,” as the employees of the adoption centers say. As Timmermans and Buchbinder put it,

The production of patients-in-waiting relates to the way screening and testing is implemented with shifting alliances between vocal patient groups, testing companies, and public health programs, combined with varying heuristic practices for interpreting results (Timmermans and Buchbinder, 2010, p. 418).

Paraphrasing Timmermans and Buchbinder (2010), I interpret adoption as an element of the management of children-in-waiting. This process implies screening and defining who is adoptable and who can adopt. These politicized diagnostic processes are implemented through the network of institutions of social care, juridical bodies, and medical authorities, as well as they are rooted in larger policies of thus far failed deinstitutionalization and the conservative pro-familia solutions. The material and ideational aspects of psychiatrization become the crucial knots in this network, within which the wellbeing of children taken away from their first-families is being acted upon.

Svend Brinkmann called a contemporary situation in which human suffering is being increasingly interpreted in terms of psychiatric conceptions and diagnostic categories as “diagnostic cultures” (Brinkmann after Nissen and Bech Risør, 2018). The moral regimes created by the infrastructure of adoption are based on psy-nomenclature, and they put much pressure on adoptive parents and children by promoting the model of individual responsibility for the possible failures. The tensions embedded in the adoption practices (Maciejewska-Mroczek and Witeska-Młynarczyk, 2022) will be actively elaborated by the state bureaucrats and the bottom-up initiatives in the upcoming decades. The psychiatric knowledge and practice will play a significant role here.

Currently, the adoptive families become patient-consumers within the system of healthcare, even though when they enter the adoption network, they start to take part in the political process of solving the social problem. They become part of the network, which enables the social problem to become privatized and the responsibility for its solution individualized (Witeska-Młynarczyk, 2022b).

## Conclusion

By bringing forward an ethnographic material from a larger study focused on the adoption practices in contemporary Poland, I meant to illustrate how the psy-knowledge and the processes of psychiatrization have become intertwined with the political process of governing children's biographies by the Polish state administering adoptions. I showed particular institutionalized forms of managing care in which various elements of psy-knowledge play an increasingly important role. In particular, the attachment theory, trauma studies, and diagnosis like RAD, FASD, or ADHD start to order the social relations between the carers, and the children and their past. The intimate practices of “kinning” are heavily intermediated by the state employees who are both bureaucrats and psy-experts. I discussed how the relationships performed among the people involved in the adoption network result in increased privatization and individualization of responsibility, as well as they lead to the strengthening of the diagnostic culture of which “adoption” is becoming a distinctive part.

## Data availability statement

The original contributions presented in the study are included in the article/supplementary materials, further inquiries can be directed to the corresponding author.

## Ethics statement

The ethical review was provided during the review process by the granting body. No special ethical review and approval was required for the study on human participants in accordance with the local legislation and institutional requirements. Written or verbal informed consent to participate in this study was provided by each participant (when relevant also by the legal guardian/next to kin).

## Author contributions

The author confirms being the sole contributor of this work and has approved it for publication.

## Funding

This paper was written based on the research funded by the National Science Center, Grant Number 2017/27/B/HS3/00645.

## Conflict of interest

The author declares that the research was conducted in the absence of any commercial or financial relationships that could be construed as a potential conflict of interest.

## Publisher's note

All claims expressed in this article are solely those of the authors and do not necessarily represent those of their affiliated organizations, or those of the publisher, the editors and the reviewers. Any product that may be evaluated in this article, or claim that may be made by its manufacturer, is not guaranteed or endorsed by the publisher.

## Footnotes

^1^I discuss the “psychiatrization” processes as they have unfolded in the Polish context in reference to children more broadly in a chapter titled “Enacting ADHD diagnosis in the landscape of care in Poland” (Witeska-Młynarczyk, 2018).

^2^This paper does not highlight the children's perspectives nor it places individual children's biographies in the center of the analysis. I discuss the psychiatrization of adoption as seen from the perspective of a minor person who was adopted in greater detail elsewhere (Witeska-Młynarczyk, 2022b).

^3^More about the research team can be found on its website: www.childhoods.uw.edu.pl.

^4^It is important to note that the research continued during COVID-19 pandemic, and the methodology had to be adjusted to the new circumstances. Approximately one-fourth of the interviews were conducted online with the use of various communicators and phone.

^5^By requiring the candidates for adoptive parents to be a married, heterosexual couple with some years of experience, the adoption centers delimit a social norm—a proper family. The detailed demands vary between adoption centers.

^6^The Supreme Audit Office reported a number of incorrections committed in this field by various adoption centres like: the actual absence of assessment, qualifying children despite the lack of needy opinions (including psychological and medical examinations), making no effort in looking for a family willing to adopt siblings and hence separating related children (Wykonywanie zadań przez ośrodki adopcyjne, 2017, p. 12).

^7^I discuss in more detail the concept of liminality in regards to adoption in another text (Witeska-Młynarczyk, 2022b).

^8^Despite the fact that more and more parents make no secret out of adoption *vis-à-vis* their children, still during this research, I met parents who did not tell their children they were adopted. This example points to the slow cultural change.

^9^The costs of institutional care for children-in-waiting are quite diverse. The calculations discussed by the psychotechnocrats at the meeting which I attended pointed to the monthly costs amounting to over 4,000 Polish zlotys per person per month. These costs grow depending on the kind of institutional care provided. The most expensive care discussed was the care provided in the so-called “therapeutic” institutions, where a psychologist and various therapists are employed. Such placement costed the state over 8,000 Polish zlotys. During the discussions, it was also mentioned that an autistic child requires 1:1 care and that the cost of such institutional care equals 12,000 Polish zlotys.

^10^When diagnosed, the prospective parents are treated with suspicion. Once this diagnostic process and the selection process are made, and the child and the adult carers are paired, the unease toward the private sphere is suspended, and the notion of “miracle” of adoption and a movement to “normalcy” are promoted. Distrust is turned into a complete trust given to the new carers as no serious supervision of the adoption process follows.

^11^I reframe the term “brokers for ADHD” coined by Philips (2006).

^12^A similar reflection was developed by Claudia Malacrida for Canada and the Great Britain in the context of ADHD, where teachers, special educators, and school psychologists “identify, assess, and administer medication to ≪problematic≫ children” (Malacrida, 2004, p. 61).

^13^The child and youth psychiatry in Poland is recognized as one of the most neglected areas of medical care. The waiting time and the accessibility of the specialist are low. While each year in Poland, a few 1,000 young people are hospitalized due to mental health problems, and an equal number of them are placed in residential care and other facilities for “troubled teens” (Golightley, 2020), there is also an alarmingly high number of youth attempting suicide (Dzieci sie licza, 2017). The media discourse depicts the system of child and youth mental healthcare as a catastrophe (Walewski, 2018; Pucułek, 2019). The journalists and experts, among others, point to the numerous shortages in staff and public resources allocated to child and youth psychiatry, as well as the unresolved emergencies and abuses, including sexual abuses and homophobic acts aimed against young people committed in their environments and spaces of care (Chotkowska and Parzuchowska, 2019; Bereś and Schwertner, 2020).
